# Glypican-4 serum levels are associated with cognitive dysfunction and vascular risk factors in Parkinson’s disease

**DOI:** 10.1038/s41598-024-54800-8

**Published:** 2024-02-29

**Authors:** Lars Tatenhorst, Fabian Maass, Hannah Paul, Vivian Dambeck, Mathias Bähr, Rosanna Dono, Paul Lingor

**Affiliations:** 1 Department of Neurology, University Medical Center Göttingen, 37099 Göttingen, Germany; 2 Center for Biostructural Imaging of Neurodegeneration (BIN), University Medical Center Göttingen, 37099 Göttingen, Germany; 3 Aix Marseille Univ, CNRS, IBDM, Turing Center for Living Systems, NeuroMarseille, 13288 Marseille, France; 4 Clinical Department of Neurology, School of Medicine, University Hospital Rechts der Isar, Technical University of Munich, 81679 Munich, Germany

**Keywords:** Glypican-4, Biomarker, Parkinson’s disease, Dementia, Vascular risk factors, Biomarkers, Neurology

## Abstract

Glypicans are biomarkers for various pathologies, including cardiovascular disease, cancer and diabetes. Increasing evidence suggests that glypicans also play a role in the context of neurodegenerative disorders. Initially described as supporting functionality of synapses via glutamate receptors during CNS development, Glypican 4 (GPC-4) also plays a role in the context of dementia via tau hyperphosphorylation in Alzheimer’s disease, which is also a co-pathology in Parkinson’s disease dementia. However, clinical evidence of circulating GPC-4 in Parkinson’s disease (PD) is missing so far. We therefore investigated GPC-4 in biofluids of PD patients. We analyzed GPC-4 levels in cerebrospinal fluid (CSF, n = 140), serum (n = 80), and tear fluid samples (n = 70) of PD patients and control subjects in a similar age range by ELISA (serum, CSF) and western blot (tear fluid). Expression of circulating GPC-4 was confirmed in all three biofluids, with highest levels in serum. Interestingly, GPC-4 levels were age-dependent, and multiple regression analysis revealed a significant association between GPC-4 serum levels and MoCA score, suggesting an involvement of GPC-4 in PD-associated cognitive decline. Furthermore, stratification of PD patients for vascular risk factors revealed a significant increase of GPC-4 serum levels in PD patients with vascular risk factors. Our results suggest GPC-4 as a clinical biomarker for vascular risk stratification in order to identify PD patients with increased risk of developing dementia.

## Introduction

Next to motor symptoms like bradykinesia, rigidity and tremor, Parkinson’s disease (PD) is also characterized by the presence of non-motor symptoms. Cognitive dysfunction, ranging from mild cognitive impairment (MCI) to Parkinson’s disease dementia (PDD), can be found in most PD patients^1^. While limbic and neocortical α-synuclein aggregates represent the main underlying pathology, tau and amyloid co-pathologies are also common in PDD^2^. Reduced amyloid-β_1-42_ levels in the cerebrospinal fluid (CSF) of patients with PD have been reported to predict cognitive decline^3^. Although α-synuclein levels in CSF of PD patients are reduced and a cortical spread of Lewy bodies can be observed in later stages of the disease, this marker cannot be used to predict cognitive dysfunction^4^. However, vascular risk factors like hypertension and heart disease are associated with cognitive decline in PD^5,6^. New biomarkers linked to this PDD related mixed pathology and/or biomarkers reflecting the vascular component of cognitive dysfunction could be helpful to monitor the progression of cognitive decline in PD.

Here, circulating glypicans might represent new potential targets. Glypicans are a family of cell surface bound heparan sulfate proteoglycans with 6 members (GPC-1 to GPC-6) described in mammals^7^. Generally, glypicans play a major role in developmental morphogenesis and are involved in the regulation of cell migration, axonal pathfinding, synaptogenesis, and structural plasticity^8^. Although being essentially membrane-linked, glypicans can be processed at the cell membrane and released as circulating protein^9^. Circulating glypicans have been proposed as fluid biomarkers for different non CNS-associated pathologies: GPC-1 can be detected in serum of patients with pancreatic cancer^10^, GPC-3 is a marker for hepatocellular carcinoma^11^ and GPC-4 has been described as a survival marker in colorectal cancer and breast cancer^12,13^, as well as for cardiovascular disease^14,15^. Furthermore, GPC-4 is also present in serum from patients with high body fat content and insulin resistance^16^. Interestingly, insulin resistance might share common dysregulated pathways with PD such as those related to abnormal mitochondrial function and glucose metabolism^17–19^. Intriguingly, insulin resistance has been described to be more common in PDD compared to PD patients without dementia^20^.

GPC-4 has further been identified as new stem cell marker and as a key regulator of signals controlling the balance between pluripotent stem cells self-renewal and differentiation^21–23^. Loss-of-GPC-4 function in pluripotent stem cells enhances differentiation of ventral midbrain dopaminergic neurons both in vitro and after brain transplantation in rat models of PD^22,24^. GPC-4 is also expressed in astrocytes, where it acts as an astrocyte-secreted protein essential for regulating synaptic activity during CNS development^25,26^. Increased glutamatergic transmission may contribute both to PD pathophysiology and to dopaminergic neuronal degeneration in a non-cell autonomous manner as an excitotoxic component^27,28^. Increasing evidence suggests that glypicans also play a role in the adult brain in the context of CNS disorders like AD, PD and ischemia^8^. Tau hyperphosphorylation as a part of an AD pathology (or co-pathology in the case of PDD) has been described recently to be directly mediated by GPC-4 due to its interaction with Apolipoprotein E4^29^. Furthermore, the amyloid precursor protein (APP), which presents the source of processed amyloid-β peptides in AD, strongly binds GPC-1^30^. In regard to PD, an interaction between APP-bound GPC-1 and α-synuclein has been discussed to moderate α-synuclein aggregation in human neural progenitor cells^31^. These interactions hint to an involvement of glypicans in AD as well as PD-related pathology, which as a mixed pathology can be found in PDD.

As clinical evidence of circulating GPC-4 in PD is missing so far, we therefore investigated circulating GPC-4 as a potential biomarker by measuring its levels in different biofluids of PD patients with well-characterized clinical profiles.

## Results

### Participants characteristics

In total, we analyzed 140 CSF samples (70 PD and 70 CTR), 80 serum samples (40 PD and 40 CTR), as well as 70 tear fluid samples (35 PD and 35 CTR). In the PD cohort, from 37 out of 40 serum samples CSF was available from the same patient. In the CTR cohort, from 35 out of 40 serum samples CSF was available from the same patient. Tear fluid was collected from separate cohorts of PD patients and CTR. None of the cohorts differed significantly for age or sex (*p* > 0.05). Demographical and clinical characteristics of the study population are given in Table 1.

**Table 1:** Demographical and clinical characteristics of the study population.

	CSF		Serum		TF	
	PD	CTR	PD	CTR	PD	CTR
	*n* = 70	*n* = 70	*n* = 40	*n* = 40	*n* = 35	n = 35
Age [years]	66.5 ± 11.3	66.6 ± 12.6	66.3 ± 11.7	64.5 ± 13.3	69.3 ± 10.3	69.9 ± 11.4
Sex [m/f]	48/22	43/27	30/10	22/18	23/13	22/14
Disease duration [years]	4.6 ± 4.1		4.1 ± 4.4		5.4 ± 5.4	
MoCA	25 ± 4.1		25 ± 3.8		24 ± 3.4	
UPDRS III	25 ± 11.9		26 ± 13.9		25 ± 11.4	
PD-NMS	7.4 ± 4.9		6.5 ± 3.8		6.6 ± 3.5	
Modified H&Y stage	2.1 ± 0.8		2.0 ± 0.7		2.3 ± 0.9	
LED	474 ± 445		450 ± 492		273 ± 298	

Data is presented as mean ± standard deviation.

CSF, cerebrospinal fluid; TF, tear fluid; PD, Parkinson's disease; CTR, control subjects with similar age range; MoCA, Montreal cognitive assessment; UPDRS III, Unified Parkinson's Disease Rating Scale III; PD-NMS, Parkinson’s disease non-motor symptoms questionnaire; H&Y, Hoehn & Yahr stage; LED, levodopa equivalent dosage.

### Glypican-4 levels in biofluids

**Figure 1:**
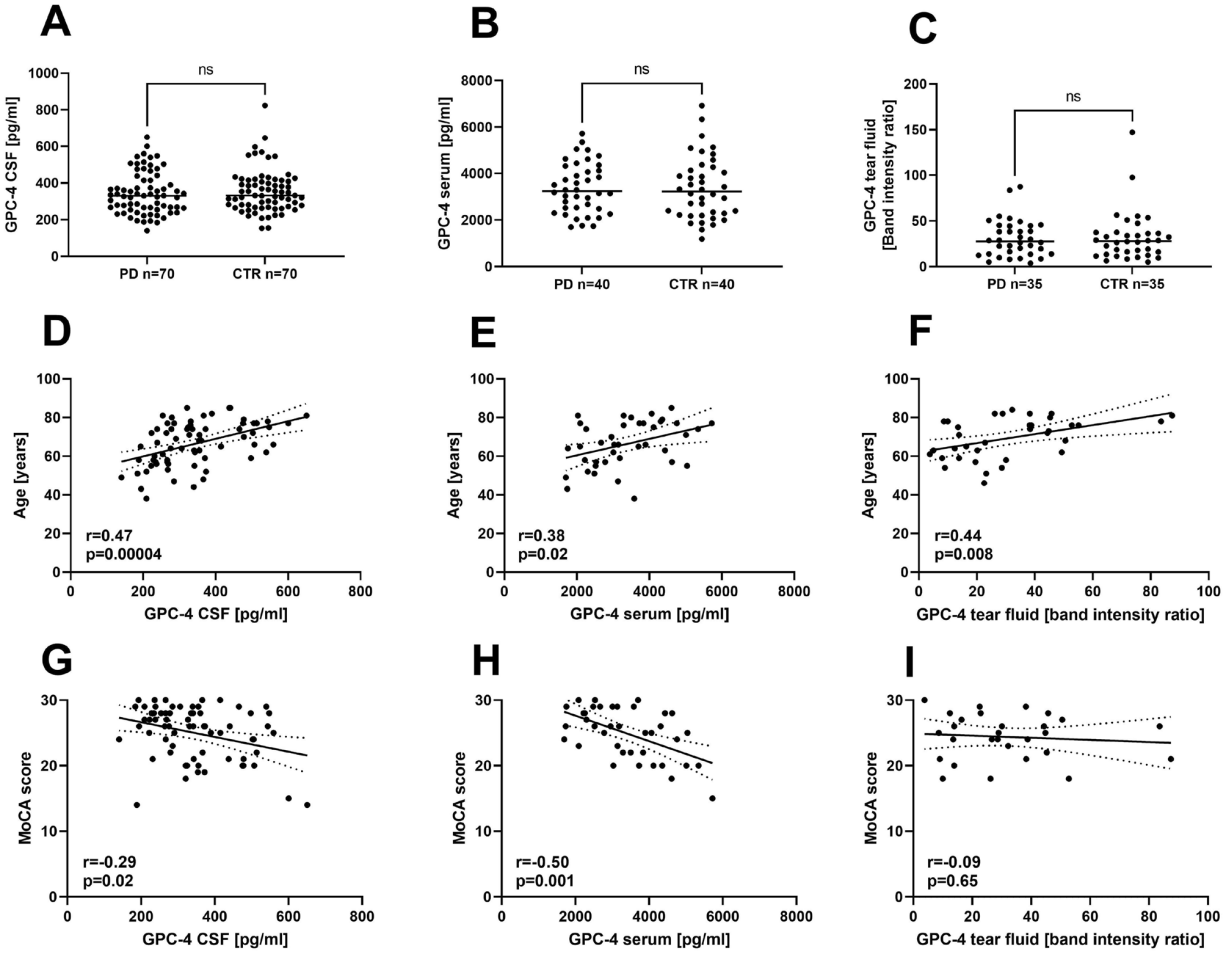
Glypican-4 levels in different biofluids and correlation with clinical parameters. (**A**–**C**) GPC-4 levels of PD patients and CTR in CSF (**A**), serum (**B**) and tear fluid (**C**). Mann–Whitney-U test, n.s., not significant. (**D**–**F**) Correlation analysis of GPC-4 levels and age of PD patients in CSF (**D**), serum (**E**) and tear fluid (**F**). (**G**–**I**) Correlation analysis of GPC-4 levels and MoCA score of PD patients in CSF (**G**), serum (**H**) and tear fluid (**I**). GPC-4, Glypican-4; PD, Parkinson’s disease; CTR, control subjects with similar age range; CSF, cerebrospinal fluid; TF, tear fluid; MoCA, Montreal cognitive assessment. r, Spearman’s Rho; ns, not significant. *p* < 0.05 was considered significant.

GPC-4 could be detected in all three biofluids tested (Fig. 1A–C). The mean GPC-4 concentration as assessed by ELISA in CSF was 345.3 ± 114.9 [range 140.4–650.8] pg/ml in PD patients versus 355.6 ± 117.5 [range 153.6–824.0] pg/ml in CTR. In serum, GPC-4 concentrations were about 10-times higher as compared to CSF, with a mean of 3371 ± 1078 [range 1696.1–5720.2] pg/ml in PD patients versus 3371 ± 1344 [range 1182.2–6920.7] pg/ml in CTR. There was a significant correlation between GPC-4 serum and CSF levels in both groups (see Supplementary Fig. 2). Due to the collection procedure of the tear fluid using RIPA buffer to elute the proteins from the Schirmer test strips, it was not possible to analyze the GPC-4 protein levels by ELISA, as the ELISA assay was not compatible with RIPA buffer. Therefore, western blot analysis was used to determine the GPC-4 protein levels in tear fluid and relative band intensity ratios were compared. The mean in the PD cohort was 30.8 [range 3.8–87.4] arbitrary units (a.u.), versus 32.1 [range 5.0–147.2] a.u. in the CTR cohort.

Interestingly, correlation with clinical parameters revealed a highly significant positive correlation of GPC-4 protein levels and age in all biofluids analyzed (Fig. 1D–F). Furthermore, we detected a significant inverse correlation of GPC-4 protein levels and cognitive function in PD assessed by the MoCA score in CSF (Fig. 1G) and serum (Fig. 1H), but not in tear fluid samples (Fig. 1I). After adjusting the GPC-4-MoCA score correlation for age as a confounder applying multiple linear regression, there was still a significant relationship between GPC-4 serum levels and MoCA score (*p* = 0.018), which could not be demonstrated for CSF (*p* = 0.32). Stratification of PD patients and CTR for different age groups revealed no significant differences between the groups (see Supplementary Fig. 3). However, the correlation of GPC-4 serum levels and MoCA score was even more significant in PD patients of older age (*p* = 0.005, see Supplementary Fig. 4). All other clinical parameters (UPDRS III, disease duration, PD-NMS, H&Y stage, LED) investigated did not result in any further significant correlations with serum or CSF levels (*p* > 0.05).

### Relationship between Glypican-4 serum levels and vascular risk factors

**Figure 2:**
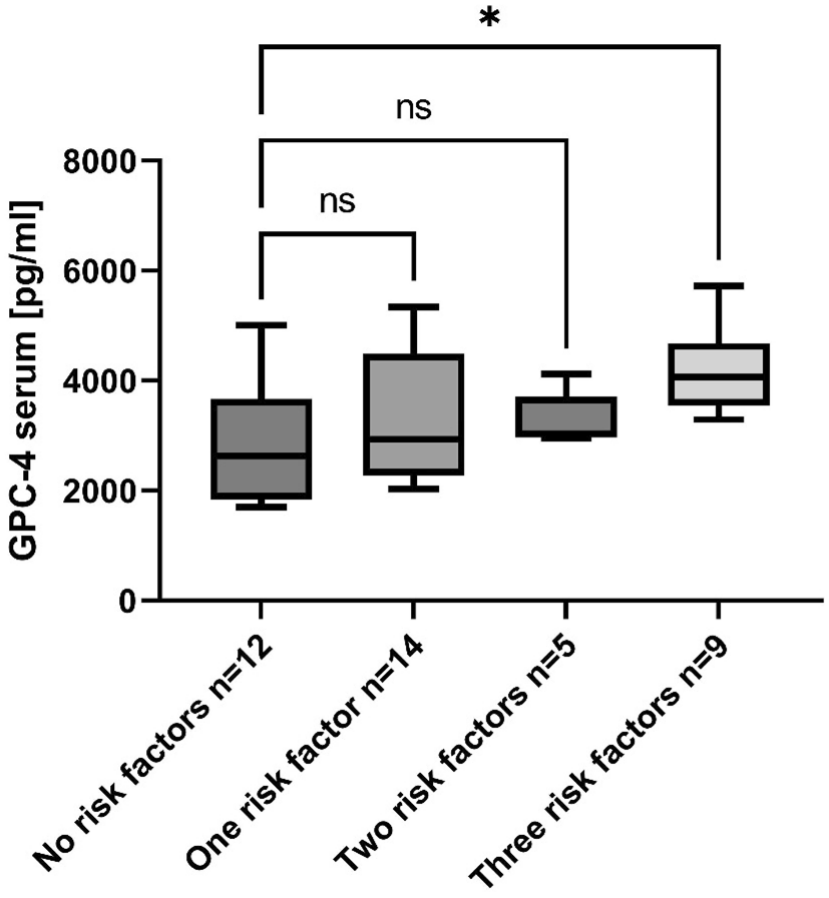
Glypican-4 serum levels in PD patients stratified for vascular risk factors (coronary artery disease, hyperlipidemia, diabetes, stroke, microangiopathy and hypertension). Stratified groups were compared applying a linear model adjusted for age and sex. *p* < 0.05 was considered significant. PD, Parkinson’s disease; GPC-4, glypican-4; ns, not significant, *p = 0.026.

GPC-4 serum levels have been described to be associated with different vascular risk factors^14,15^, therefore association between GCP-4 serum levels and vascular risk factors was evaluated in the PD group. Following parameters were considered: coronary artery disease (n = 6), hyperlipidemia (n = 10), diabetes (n = 6), stroke (n = 4), microangiopathy (n = 2) and hypertension (n = 23). Information on nicotine abuse were not available. GPC-4 serum levels and age distribution of the respective vascular risk factor groups are depicted in supplementary Fig. S1. PD patients were further stratified for no, one, two or three vascular risk factors and a linear model adjusted for age and sex was applied to compare groups. A significant difference could be found between GPC-4 serum levels in PD patients without vascular risk factors and patients with three risk factors (*p* = 0.026; Fig. 2).

## Discussion

To the best of our knowledge, this is the first description of circulating GPC-4 in Parkinson’s disease. Interestingly, GPC-4 serum levels in our study showed a wide abundance range in PD patients as well as CTR and were largely overlapping, which did not allow for a separation of both groups. In a recent study investigating the role of GPC-4 in pregnancy with relation to diabetes^32^, the GPC-4 serum levels were in a similar range as the results presented here, showing slightly higher values in our study. This could be explained by the younger age of subjects included in the study with pregnant women^32^ and goes in line with our finding that circulating GPC-4 protein levels show a significantly positive correlation with age in all three biofluids tested. Recent analyses of serum GPC-4 in patients with cardiovascular disease corroborate this correlation^14,15^.

CSF levels of circulating GPC-4 protein were about ten times lower as compared to the serum levels, therefore the main expression can be found outside the central nervous system, where GPC-4 is highly expressed in adipocytes^16^. However, at least part of GPC-4 in the CSF can be CNS-derived, for example it can be released from astrocytes^29^. The only glypican described in CSF so far is GPC-2, and was proposed as a marker of neurogenesis^33^. Lugert and colleagues further analyzed the protein levels of GPC-2 and GPC-4 in neural progenitor cells during differentiation, where GPC-2 protein level increased over time, whereas GPC-4 level peaks after two weeks of neuronal differentiation. However, they did not further investigate GPC-4 levels in CSF^33^.

Interestingly, we were able to quantify GPC-4 also in tear fluid. Tear fluid (TF) is mainly produced by lacrimal gland, ocular superficial epithelial cells, Meibomian glands, and stromal immune cells^34^. More than 1500 different proteins can be detected^35^, making it a valuable source for protein analysis. Recently, changes in tear fluid composition have been reported in neurodegenerative disorders like PD and AD^36,37^, but GPC-4 was not yet investigated. Comparable with serum and CSF results, GPC-4 in TF could not be used to discriminate PD patients and CTR.

Importantly, we detected an inverse correlation between MoCA score values of PD patients and corresponding GPC-4 levels in serum and CSF, which was still present in serum after adjusting for age applying multiple linear regression analysis. This significant correlation between circulating GPC-4 levels in serum and the corresponding MoCA score indicates a potential role of GPC-4 in PD-associated cognitive decline, which appears even more prominent in PD patients of older age. This was further corroborated by analyzing different age groups based on the mean age of 66 years in the PD group in this study. While no significant correlation could be detected in younger PD patients, a highly significant correlation was observed in PD patients older than 66 years. Due to the fact that the incidence of vascular risk factors generally increases with age, one can assume that the lower incidence of vascular risk factors in younger PD patients is not yet sufficient to establish a significant correlation between MoCA score and GPC-4 levels. Additionally, cognitive dysfunction in younger individuals presumably may be rather associated with non-vascular mechanisms related to the accumulation of α-synuclein or even amyloid-β protein^38^. Therefore, analysis of GPC-4 may be more suitable in patients of higher age with different vascular risk factors, potentially indicating GPC-4 as an additional vascular factor in cognitive dysfunction.

Decades ago, glypican has already been described as a mediator of amyloid-β toxicity^39^. Just recently, Saroja and colleagues showed that astrocyte-secreted GPC-4 is involved in tau hyperphosphorylation in AD^29^. As AD-related pathology also contributes to the cognitive dysfunction in PD^38^, GPC-4 might play a role in the underlying pathology in PDD. However, since in this study a relationship between GPC-4 and MoCA score could only be confirmed in serum but not in CSF, we assume that GPC-4 serum levels may be reflecting vascular pathology associated with cognitive decline. GPC-4 is a part of the endothelial glycocalyx, which maintains the integrity of the border between blood flow and endothelium under physiological conditions^40^, and is degraded with advanced age as well as under inflammatory conditions, making GPC-4 a marker for vascular risk and mortality^14^. Serum GPC-4 has been shown to be associated with different vascular risk factors like increased age, body mass index, brain natriuretic peptide, and oxidized low density lipoprotein, as well as insulin resistance^14,20^. Strikingly, vascular risk factors also affect attention and executive functions in PD^6^. Therefore, we hypothesize that serum GPC-4 could reflect the overall vascular risk in PD patients, which is per se an age-dependent risk factor for cognitive decline in different forms of dementia including PDD^6,41^. We suggest that the potential of GPC-4 as a marker for vascular risk and/or cognitive dysfunction is not specific for PD, but may also be useful in other conditions associated with cognitive decline. Multicentric trials including Alzheimer’s disease, vascular dementia, frontal lobe dementia and dementia with Lewy bodies will be needed to target this question.

The correlation of GPC-4 serum levels and MoCA score values in our study holds the potential to use serum GPC-4 as a molecular biomarker for vascular risk stratification in order to identify PD patients with increased risk of developing dementia, presumably of higher age. Future longitudinal clinical studies are warranted to validate the potential of GPC-4 for dementia tracing in neurodegenerative disorders.

## Methods

### Study participants

PD patients diagnosed according to MDS criteria^42^, as well as control subjects with similar age range (CTR) without signs of neurodegenerative, neuroinflammatory, neurooncological or acute ischemic central nervous diseases were included in this study. All PD patients underwent neurological examination by movement disorder specialists, including the assessment of motor (Unified Parkinson’s Disease Rating Scale, part III [UPDRS III] and Hoehn & Yahr stage) and cognitive function (Montreal Cognitive Assessment [MoCA]). Non-motor symptoms were evaluated using the Parkinson’s Disease Non-Motor Symptoms Questionnaire (PD-NMS). The levodopa equivalent dose (LED) was calculated according to Tomlinson et al., 2010^43^.

### Biofluid collection and analysis

Biosamples were obtained from the biobank of the Department of Neurology of the University Medical Center Göttingen, Germany. CSF and serum sample collection was performed according to standard biosampling protocols^44^. Samples were centrifuged and aliquots were stored at − 80 °C within 1–2 h. Tear fluid was collected as described before^45^. Briefly, we used standard Schirmer test strips that were applied into the inferior-temporal aspect of the conjunctival sac of both eyes. After collection of tear fluid, strips from both eyes were pooled in polypropylene reaction tubes and immediately frozen in liquid nitrogen. Subsequently, the strips were stored at − 80 °C until further analysis.

To assess GPC-4 levels in CSF and blood serum of PD patients and CTR we used a commercial ELISA assay with highly specific human GPC-4 antibodies (#SEA998Hu, Cloud-Clone Corp., Katy, TX). In a preliminary investigation, we detected GPC-4 in undiluted samples at very high levels, especially in blood serum where the upper detection limit of the ELISA kit was exceeded. To remain in the optimal detection range, CSF samples were diluted 1:2 in PBS and serum samples were diluted 1:7, respectively. All samples were analyzed as technical triplicates and normalized to an internal protein standard according to manufacturer’s instructions. Internal controls were analyzed on each ELISA plate to ensure inter assay comparability.

Total tear fluid protein content was eluted from the test strips with RIPA buffer with proteinase and phosphatase inhibitors cocktail (cOmplete and PhosSTOP, Merck, Darmstadt, Germany), and centrifuged at 16,000 × g for 30 min at 4 °C. Protein yield was determined by BCA assay. Western blot analysis was performed using anti-GPC-4 rabbit-antibody (Proteintech #13048-1-AP, Rosemont, PA) and secondary HRP-coupled anti-rabbit antibody (Cell signaling #7074, Frankfurt, Germany). Western blots were quantified using a Fusion FX analysis system and Evolution Capt software (Vilber, Eberhardzell, Germany) and normalized to a pooled TF protein standard from healthy controls.

### Statistical analysis

Statistical analysis was performed using GraphPad Prism 9.4.0 software or the R software version 4.3.1. Protein quantification of PD patients and CTR was compared as nonparametric data using Mann–Whitney U test. The sex distribution of the two cohorts was compared as qualitative data using the Chi-Square test. Clinical data included age, sex, disease duration, UPDRS III score, Hoehn & Yahr stage, MoCA score, PD-NMS score and LED. Spearman correlation was performed to detect correlations between GPC-4 levels and clinical data. Correlation between GPC-4 levels and MoCA score was adjusted for age using multiple linear regression. PD patients stratified for vascular risk factors were compared applying a linear model adjusted for age and sex. The linear model was also used to compare PD patients and CTR stratified for different age groups, based on the mean age of 66 years in the PD group. *p* < 0.05 was considered significant.

### Ethical approval and consent to participate

A permission of the local ethics committee has been obtained prior to the initiation of the study (Ethics committee of the University Medicine Göttingen, No. 13/11/12). Written consent was provided by all patients or care givers. The study conforms to the Code of Ethics of the World Medical Association (Declaration of Helsinki).

### Supplementary Information

Supplementary Information.

## Data Availability

The datasets used and/or analyzed during the current study are available from the corresponding author on reasonable request.
