# Supplementary material for: Glypican-4 serum levels are associated with cognitive dysfunction and vascular risk factors in Parkinson’s disease
Source: Sci Rep. 2024 Feb 29;14:5005. doi: 10.1038/s41598-024-54800-8 (PMC10904781; doi:10.1038/s41598-024-54800-8)
Supplement: Supplementary file 1 — Supplementary Information. [file 41598_2024_54800_MOESM1_ESM.docx]

Scientific Reports

**Glypican-4 serum levels are associated with cognitive dysfunction and vascular risk factors in Parkinson’s disease**

**Lars Tatenhorst^1,2,+^, Fabian Maass^1,+^, Hannah Paul^1,2^, Vivian Dambeck^1,2^, Mathias Bähr^1,2^, Rosanna Dono^3,+^, and Paul Lingor^1,2,4,+,*^**

^1^Department of Neurology, University Medical Center Göttingen, Göttingen, 37099, Germany

^2^Center for Biostructural Imaging of Neurodegeneration (BIN), University Medical Center Göttingen, Göttingen, 37099, Germany

^3^Aix Marseille Univ, CNRS, IBDM, Turing Center for Living Systems, NeuroMarseille, Marseille, 13288, France

^4^Clinical Department of Neurology, School of Medicine, University Hospital rechts der Isar, Technical University of Munich, Munich, 81679, Germany

^*^corresponding author: [paul.lingor@tum.de](mailto:paul.lingor@tum.de)

^+^these authors contributed equally to this work

**Additional information**

Supplementary data


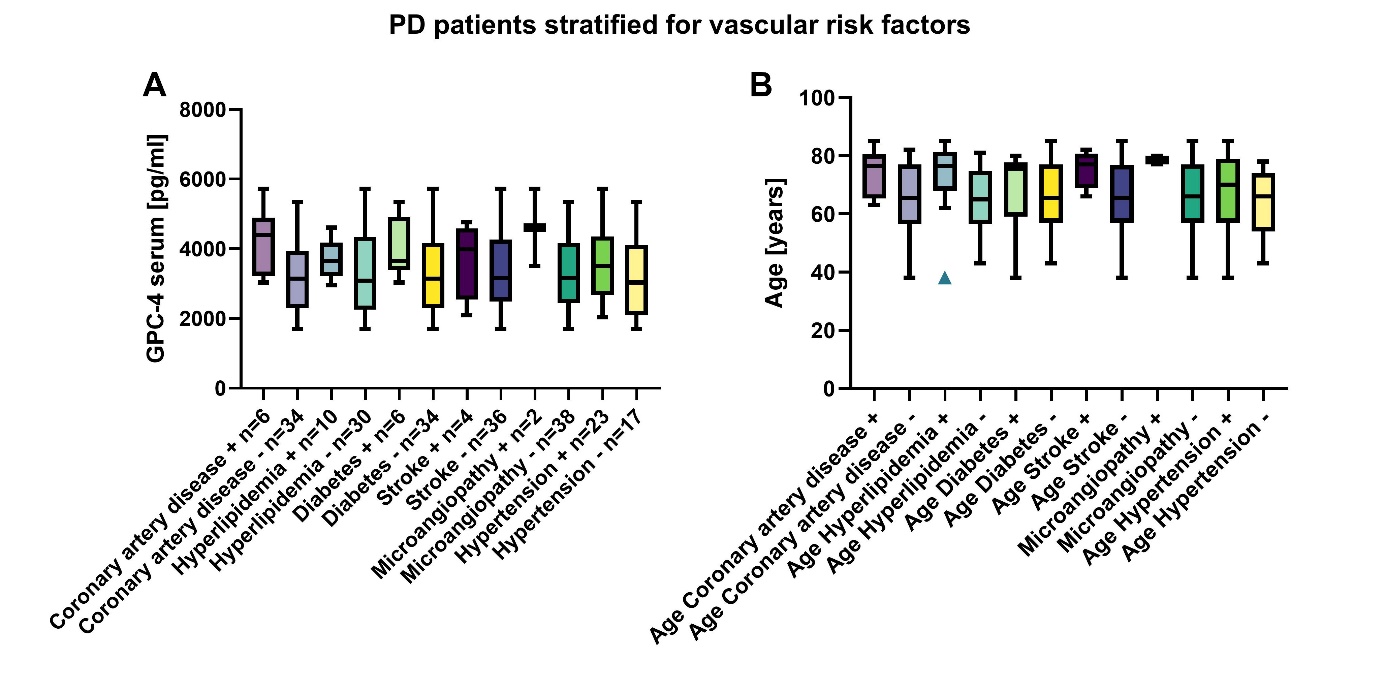


**Supplementary figure 1.** PD patients stratified for vascular risk factors (coronary artery disease, hyperlipidemia, diabetes, stroke, microangiopathy and hypertension). A: GPC-4 serum levels in PD patients according to vascular risk factors. B: Age distribution in PD patients according to vascular risk factors. PD = Parkinson’s disease, GPC-4 = Glypican-4.


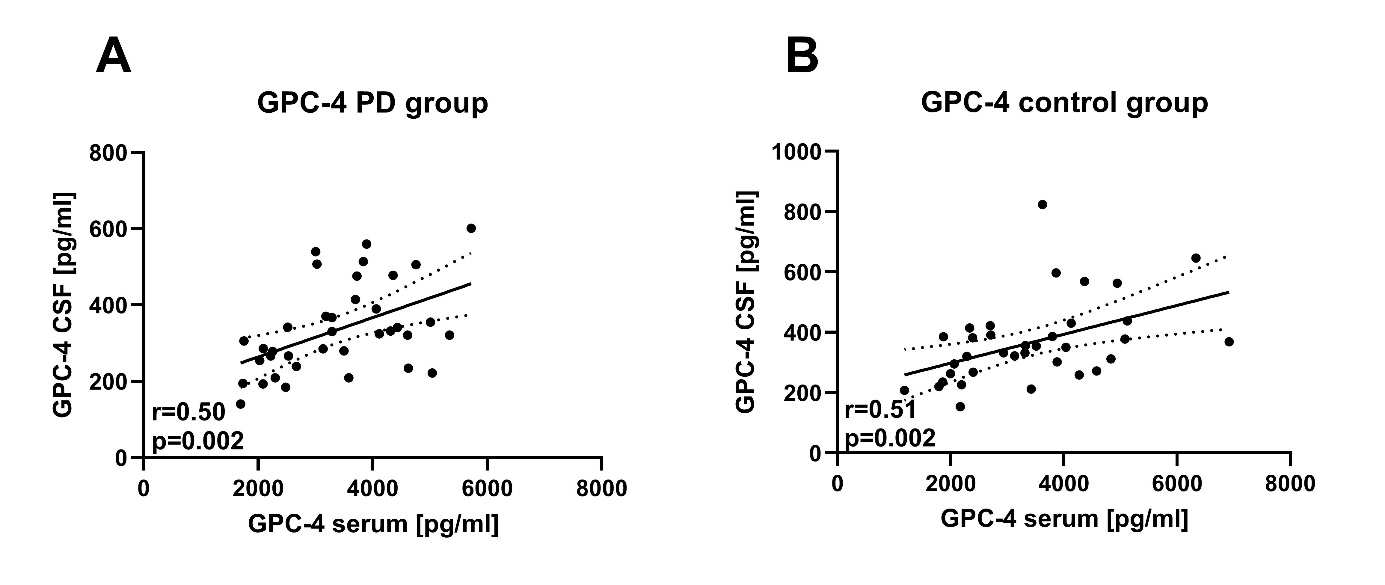


**Supplementary figure 2.** Spearman correlation analysis showing a significant correlation between corresponding GPC-4 serum and CSF levels in both PD patients (A) and controls (B), respectively. PD = Parkinson’s disease, GPC-4 = Glypican-4, CSF = cerebrospinal fluid, r = Spearman’s Rho, p < 0.05 was considered significant.


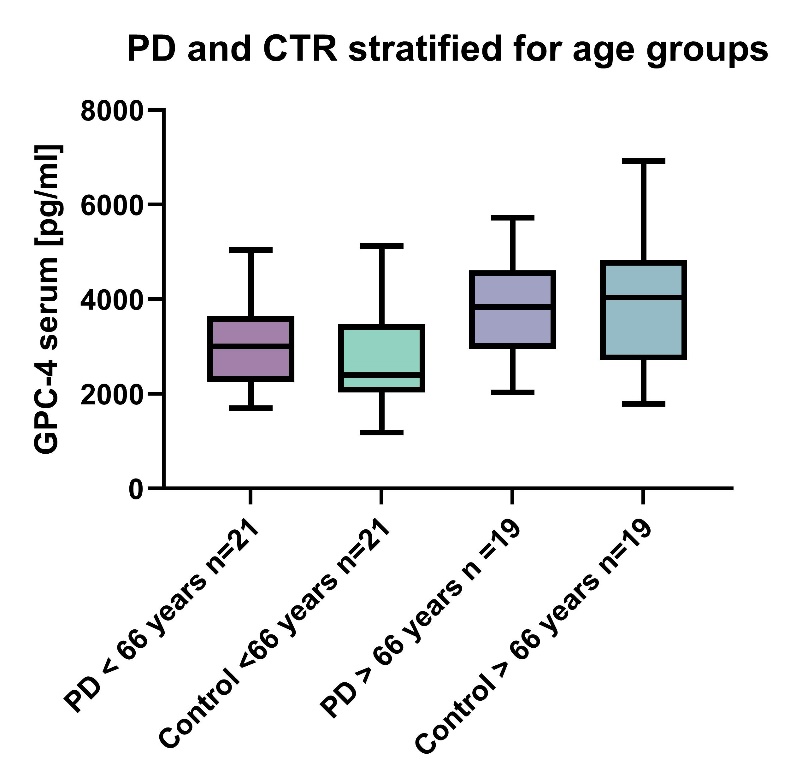


**Supplementary figure 3.** GPC-4 serum levels of PD patients and controls stratified for age, according to the mean age of the respective groups (see Table 1). There were no significant differences between the groups. PD = Parkinson’s disease, CTR = controls, GPC-4 = Glypican-4.


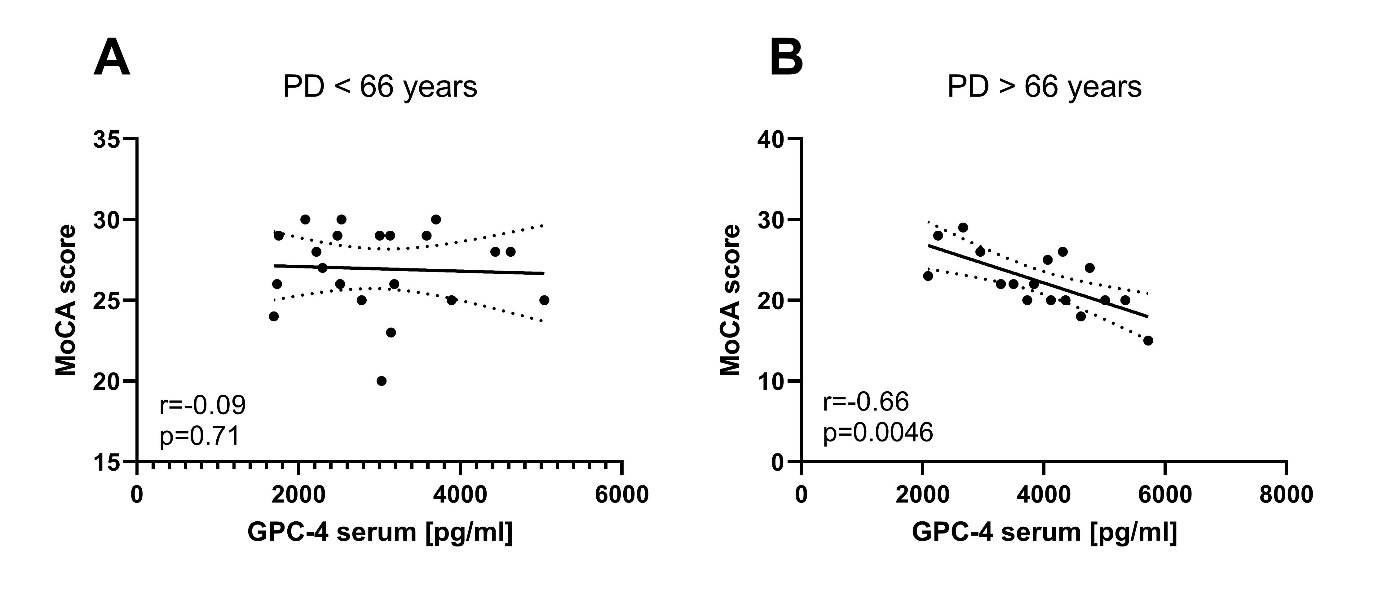


**Supplementary figure 4.** Spearman correlation analysis of corresponding GPC-4 serum level and MoCA score in PD patients stratified for different age, according to the mean age of the respective groups (see Table 1). Whereas no significant correlation was detected in PD patients younger than 66 years (A), PD patients older than 66 years show a highly significant correlation (B). PD = Parkinson’s disease, GPC-4 = Glypican-4, MoCA = Montreal Cognitive Assessment, r = Spearman’s Rho, p < 0.05 was considered significant.

**Supplementary table 1.** Spearman correlation analysis showing a significant correlation between age and the corresponding GPC-4 levels in serum as well as CSF in both PD patients and controls, respectively. PD = Parkinson’s disease, CTR = control group, GPC-4 = Glypican-4, CSF = cerebrospinal fluid, r = Spearman’s Rho, CI = confidence interval, p < 0.05 was considered significant.

|  | **PD** | **CTR** | **PD** | **CTR** |
| --- | --- | --- | --- | --- |
|  | **GPC-4 in CSF vs. age** | | **GPC-4 in serum vs. age** | |
| **Spearman’s Rho** | r=0.4677 | r=0.3575 | r=0.3837 | r=0.516 |
| **95% CI** | 0.2548 – 0.6373 | 0.1268 – 0.5515 | 0.0725 – 0.6268 | 0.2347 – 0.7176 |
| **p value (two-tailed)** | p<0.0001 | p=0.0024 | p=0.0145 | p=0.0007 |
